# Genome guided investigation of antibiotics producing actinomycetales strain isolated from a Macau mangrove ecosystem

**DOI:** 10.1038/s41598-018-32076-z

**Published:** 2018-09-24

**Authors:** Dini Hu, Yan Chen, Chenghang Sun, Tao Jin, Guangyi Fan, Qiwen Liao, Kai Meng Mok, Ming-Yuen Simon Lee

**Affiliations:** 1 Faculty of Science and Technology, Department of Civil and Environmental Engineering, University of Macau, Macao, China; 2 State Key Laboratory of Quality Research in Chinese Medicine and institute of Chinese Medical Sciences, University of Macau, Macao, China; 3 Institute of Medicinal Biotechnology, Chinese Academy of Medical Science & Peking Union Medical College, Tiantanxili No 1, Beijing, 100050 P.R. China; 4 Beijing Genome Institute–Shenzhen, Shenzhen, 518083 China

## Abstract

Actinomycetes are a heterogeneous group of gram positive filamentous bacteria that have been found to produce a wide range of valuable bioactive secondary metabolites, particularly antibiotics. Moreover, actinomycetes isolated from unexplored environments show an unprecedented potential to generate novel active compounds. Hence, in order to search for novel antibiotics, we isolated and characterized actinomycetes strains from plant samples collected from a mangrove in Macau. Within the class of actinobacteria, fourteen actinomycetes isolates have been isolated and identified belonging to the genus of *Streptomyces*, *Micromonospora, Mycobacterium, Brevibacterium, Curtobacterium* and *Kineococcus* based on their 16S rRNA sequences. Further whole genome sequencing analysis of one of the isolated *Streptomyces* sp., which presented 99.13% sequence similarity with *Streptomyces parvulus* strain 2297, showed that it consisted of 118 scaffolds, 8,348,559 base pairs and had a 72.28% G + C content. In addition, genome-mining revealed that the isolated *Streptomyces* sp. contains 109 gene clusters responsible for the biosynthesis of known and/or novel secondary metabolites, including different types of terpene, T1pks, T2pks, T3pks, Nrps, indole, siderophore, bacteriocin, thiopeptide, phosphonate, lanthipeptide, ectoine, butyrolactone, T3pks-Nrps, and T1pks-Nrps. Meanwhile, the small molecules present in ethyl acetate extract of the fermentation broth of this strain were analyzed by LC-MS. Predicted secondary metabolites of melanin and desferrioxamine B were identified and both of them were firstly found to be produced by the *Streptomyces parvulus* strain. Our study highlights that combining genome mining is an efficient method to detect potentially promising natural products from mangrove-derived actinomycetes.

## Introduction

Secondary metabolites, also referred to as natural products, are small, organic molecules that have diverse and often very potent biological activities^1^. Actinomycetes has been extremely useful to the medical industry due to their astonishing ability to produce secondary metabolites with diverse antimicrobial activities and complex chemical structures^2^. Actinomycetes are usually isolated from natural soil environment^3^, and many antibiotics and secondary metabolites have been derived from them and used extensively^4,5^. However, in recent years, the chances of discovering completely novel natural products from known actinomycetes strains have reduced^6^, which means that we need to focus on the isolation of actinomycetes strains from new, unexplored or extreme environments, such as the marine environment^7–9^.

Mangroves, which are important inter-tidal estuarine wetlands along coastlines of tropical and subtropical regions, are often situated in areas of high anthropogenic influence, being exposed to pollutants^10^. The mangrove has recently been demonstrated to be an ecosystem with many unique forms of actinomycetes due to its sediment properties of anaerobic condition, and due to being rich in sulphide, with high salinity and organic matter content^11^. Such conditions are extremely different from terrestrial conditions, such that microbial living there, especially actinomycetes, have distinctive characteristics from terrestrial actinomycetes and therefore might have the potential to produce special or unknown bioactive metabolites^12,13^. This unique adaptation characteristic of actinomycetes could serve a source of important or novel natural products^14^. Previous research supports this viewpoint and it has been demonstrated that actinomycetes from mangrove can produce novel types of new secondary metabolites^15^. Many secondary metabolites have also been obtained from mangrove actinomycetes strains and possess immense biological activities^16^.

Molecular ecological studies on microbial communities from mangrove environments revealed the presence of a rich diversity of actinomycetes taxa^17,18^. Compared to traditional cultivation methods, an improved strategy is searching for secondary metabolites by combining DNA technology for capturing genes and complete pathways of secondary metabolites producers^19^. Approximately 50% of actinomycetes strains are from the genus *Streptomyces*, and about 75% of commercially useful antibiotics are derived from this genus^20^. The first complete genome of the model strain of *Streptomyces coelicolor* have was in 2002 and revealed unprecedented potential to synthesize antibiotic compounds previously undetected by traditional cultivation, extraction and bioactivity testing^21^. To date, several strains of *Streptomyces* have been characterized and are publicly available^22–24^. Therefore, sequencing actinomycetes strains from mangroves may provide insight for the discovery of novel secondary metabolites. Moreover, genome-guided investigation of secondary metabolites may also help us to detect biosynthesis genes that cannot be expressed, or are expressed at a very low level, in laboratory conditions^25^.

Macau mangrove forest is a unique habitat within a tropical and subtropical tidal area. It is located on the Pearl River Delta, which is vulnerable to developments in the area and is also a heavily exploited ecological niche. The aim of this study was (1) to isolate actinomycetes strain from mangrove plants using a culturable method and to identify the isolates using 16S rRNA sequences; (2) to use whole-genome sequencing to detect the biosynthetic gene cluster and enzymes related to antibiotic production; and (3) to do a preliminary analysis of small molecule compounds as fermentation products of LC-MS. With this study, we hoped to discover some prominent antibiotics candidates for further research and applications in the medical industry.

## Materials and Methods

### Environmental sampling

Plant samples were collected from a mangrove forest located in Macau, China from March 2017 to May 2017. Samples were collected from three different mangrove trees, including *Acanthus ilicifolius, Aegiceras corniculatum* and *Kandelia candel*. Samples were collected at three different sites, including a high salt environment (1) (22°8′30′′N 113°33′11′′E), a coastal area (2) (22°8′29′′N 113°33′5′′E) and a polluted environment which is adjacent to a sewage plant (3) (22°7′51′′N 113°33′7′′E) (Table 1). *Kandelia candel* was only collected from area (2), because of which not found in the high salt and polluted environments while only *Aegiceras corniculatum* could be found near the sewage plant. Collected plant specimens were placed into sterile plastic bags and immediately transported to the laboratory.

**Table 1:** Source of plant materials collected from mangrove forest.

Sampling sites	Coordinate	Samples
1	22°8′30′′N 113°33′11′′E	Aegiceras corniculatum, Acanthus ilicifolius
2	22°8′29′′N 113°33′5′′E	Aegiceras corniculatum, Kandelia candel, Acanthus ilicifolius
3	22°7′51′′N 113°33′7′′E	Aegiceras corniculatum

### Selective isolation of Actinomycetes

The plant samples were surface-cleaned with 1% Tween-20 for 1 min, NaClO (0.4%) for 8 min, 2.5% NaS_2_O_3_ for 10 min, 75% ethanol for 7 min and 10% NaHCO_3_ for 10 min^26^. The cleaned samples were smashed by adding 50 mL sterile PBS buffer. After dilution into 10^−4^, fractions (100 uL) preparations were plated onto isolation plates.

Dilutions of suspensions of each sample were spread onto seven different types of isolation medium: ISP media 2 (containing, per liter distilled water: 4.0 g yeast extract, 10.0 g malt extract, 4.0 g dextrose, 20.0 g agar, pH 7.2)^27^, ISP media 4 (containing, per liter distilled water: 10.0 g starch, 1.0 g K_2_PHO_4_, 1.0 g MgSO.6H_2_O, 1.0 g NaCl, 2.0 g (NH_4_)_2_SO_4_, 2.0 g CaCO_3_, 0.001 g FeSO_4_.7H2O, 0.001 g MnCl_2_.4H_2_O, 0.001 g ZnSO_4_.7H_2_O, 20.0 g agar, pH 7.2)^28^, ISP media 7 (containing, per liter distilled water: 1.0g l-asparagine, 0.5g l-tyrosine, 0.5 g K_2_HPO_4_, 0.5 g MgSO_4_.7H_2_O, 0.5 g NaCl, 20 g agar, pH 7.2)^22^, Gauze No.1 (conraining, per liter distilled water: 20.0 g solube starch, 0.5 g sodium chloride, 0.01 g ferrous sulfate, 1.0 g potassium nitrate, 0.5 g dipotassium hydrogen phosphate, 0.5 g magnesium sulfate, 15.0 g agar, pH 7.2)^29^, Nutrient Agar (containing, per liter distilled water: 10.0 g peptone, 3.0 g beef extract, 5.0 g sodium chloride, 15.0 g agar, pH 7.2)^30^, halothiobacillus HL2 medium (containing, per liter water: 10.0 g glucose, 5.0 g peptone, 3.0 g tryptone, 5.0 g NaCl, 20.0 g agar, pH 7.2)^31^ and Czapek (containing, per liter distilled water: 3.0 g sodium nitrate, 1.0 g dipotassium hydrogen phosphate, 0.5 g magnesium sulfate, 0.5 g potassium chloride, 0.01 g ferrous sulfate, 30.0 g sucrose, 15.0 g agar, pH 7.2)^32^. All mediums were supplemented with potassium dichromate (100 mg/L) and incubated at 28 °C for 7–30 days^33^. Purified cultures were maintained on ISP media 2 and isolates were conserved at 4 °C for short-term storage, and as glycerol suspensions (20%, v/v) at −20 °C for long-term storage.

### Extraction of DNA from pure cultures and PCR amplification of 16S rRNA

The DNA of isolated colonies were extracted to identify the bacteria species. Each culture was suspended in 50 mL Chelex-100 buffer and boiled for 15 min at 99 °C and 650 rpm. The resultant preparations were centrifuged at 17,000 g for 30 min at room temperature. The upper layers, which contained DNA, were transferred to new tubes and used as DNA template. The DNA yield and quality were assessed using 1.0% (w/v) agarose gel electrophoresis. The primer pair 27 F (5′-AGAGTTTGATCCTGGCTCAG-3′) and 1492 R (5′-TACGGCTACCTTGTTACGACTT-3′) were used for PCR amplification^34^. The PCR reaction was performed in a final volume of 50 μL, which was composed of template DNA (1 μl upper aqueous layer), 1.5 mM MgCl_2_, 0.2 mM of each dNTP, 200 pM of primer, and 2U of Taq polymerase with the appropriate reaction buffer under the following conditions: initial denaturation at 95 °C for 5 min, followed by 35 cycles of 94 °C for 1 min, annealing at 55 °C for 1 min, and 72 °C for 2 min. The amplification products were separated by gel electrophoresis in 1% agarose gel. The products were then sequenced using the Sanger sequencing platform.

### Genome sequencing, assembly and annotation

Genomic DNA was isolated using the TIANamp Bacteria DNA Kit (TIANGEN Biotech Co. LTD). The genomic DNA library was constructed using the NEBNext Ultra II DNA Library Prep Kit for Illumina sequencing. The library was sequenced using an Illumina NovaSeq HiSeq. 4000. Genome assembly of the pooled sequencing reads was performed by IBDA, MetaGeneMark was used for gene prediction. Transfer RNAs (tRNAs) were predicted by tRNAscan-SE and ribosomal RNAs (rRNAs) by rnammer. Functional categories were assigned by searching against the Kyoto Encyclopedia of Genes and Genomes (KEGG) database.

### Phylogeny and genome mining

Mega 7.0 was used to demonstrate an evolutionary phylogenetic relationship among different species; 16S rRNA sequences of *Streptomyces* sp.03 was retrieved from the annotation results. In our study, ten relevant actinomycetes species were selected, the sequences of which were downloaded from the NCBI database to further verify the branches.

These species are *Streptomyces coelicolor* A3(2) (Biosample accession: X60514.1, Coelichelin producer), *Actinoplanes friuliensis* HAG010964 (Biosample accession: NR_104746.1, Friulimicin producer), *Streptomyces* sp. SCSIO 01127 (Biosample accession: JF794566.1, Lobophorin producer), *Streptomyces viridochromogenes* (Biosample accession: FJ790787.1, Laspartomycin producer), *Streptomyces kanamyceticus* strain BCCO 10_900 (Biosample accession: KP718566.1, Kanamycin producer), *Streptomyces griseoflavus* strain SAEM-16 (Biosample accession: LC150532.1, Colabomycin producer), *Streptomyces parvulus* strain NBRC 13193 T (Biosample accession: KY777591.1), *Streptomyces parvulus* strain 13193 T (Biosample accession: KY771080.1), *Streptomyces parvulus* strain SPS-W1 (Biosample accession: KY458978.1), and *Streptomyces parvulus* strain K-15 (Biosample accession: KY038196.1).

The online software of anti-SMASH was used to predict the gene clusters and secondary metabolites (https://antismash.secondarymetabolites.org/#!/about). The assembled scaffolds of the *Streptomyces* sp.03 were submitted to the anti-SMASH server to search for potential secondary metabolite biosynthetic gene clusters. The core structures of secondary metabolites biosynthetic gene clusters were identified by anti-SMASH and extracted for comparison with known gene clusters of other species using BLAST.

### Crude extract preparation and mass spectrometric analysis

ISP media 2 was used as the fermentation medium. The purified isolates were transferred to a 50 mL centrifuge tube containing 20 mL of the fermentation medium and cultured at 250 rpm, for 7 days at 28 °C. Crude extracts were prepared by adding 60 mL ethyl acetate to the cultures, and the fraction of the resultant extracts were dried at 60 °C, dissolved in 3 mL methanol and used in the biochemical screen. An 4000 Q TRAP LC/MS/MS system was interfaced with the mass spectrometer for secondary metabolites separation and analysis by micro-ESI-MS. Full-scan data was acquired in the positive ion mode from 100 to 1000 m/z at an flow rate of 0.6 s per spectrum through the MS analysis. ESI source were set as follows: capillary voltage of 3.0 kV, sample cone voltage of 20 V. The metabolic profiling including the chromatographic peak was identified by comparing with the Human Metabolome Database (HMDB)^35^ (http://www.hmdb.ca/).

## Results

### Isolation and identification of actinomycetes strains

In total, 71 bacteria isolates were isolated from plant samples, among which 14 isolates were identified as belonging to the class of actinobacteria (Supplementary Table S1 and Table 2). Isolates were identified using PCR and sequencing of 16S rRNA gene sequences. Results showed that they belong to six genera in class of actinobacteria, including *Streptomyces, Micromonospora, Mycobacterium, Microbacterium, Kineococcus* and *Brevibacterium*. All the actinomycetes strains were isolated from seven isolation mediums, namely ISP2, ISP7, Nutrient agar, Gauze No.1 and Czapek. These results indicated that Gauze No.1 was the most suitable medium for isolation of actinomycetes strains from plant samples and most of the actinomycetes strains were isolated from *Kandelia candel*. Among them, one *Streptomyces* sp.03 which show 99.13% 16S similarity with *Streptomyces parvulus* was selected for further genome analysis and secondary metabolite identification.

**Table 2:** Isolated actinobacterial community from the plant samples based on the 16S rRNA sequences.

Sampling Date	Sampling site	Tree	Plates	Top-hit taxon at species level	Similarity based on 16S (%)
2017.03	3	Aegiceras corniculatum	Gause No. 1	*Micromonospora aurantiaca* sp. 01	99.28
2017.03	3	Aegiceras corniculatum	Gause No. 1	*Streptomyces ederensis* sp. 02	98.79
2017.03	2	Kandelia candel	Gause No. 1	*Streptomyces parvulus* sp. 03	99.13
2017.03	2	Kandelia candel	Gause No. 1	*Streptomyces hyderabadensis* sp. 04	99.18
2017.05	2	Kandelia candel	Czapek	*Streptomyces olivaceus* sp. 05	99.25
2017.05	2	Kandelia candel	Czapek	*Streptomyces pactum* sp. 06	99.57
2017.05	2	Kandelia candel	Nutrient Agar	*Mycobacterium saopaulense* sp. 07	99.90
2017.05	2	Kandelia candel	Gause No. 1	*Streptomyces cuspidosporus* sp. 08	98.28
2017.05	2	Acanthus ilicifolius	ISP2	*Microbacterium hydrothermale* sp. 09	98.58
2017.05	1	Aegiceras corniculatum	ISP7	*Kineococcus aurantiacus* sp. 10	98.20
2017.05	2	Aegiceras corniculatum	Nutrient Agar	*Brevibacterium sediminis* sp. 11	98.90
2017.05	2	Acanthus ilicifolius	Nutrient Agar	*Kineococcus mangrovi* sp. 12	98.00
2017.05	2	Aegiceras corniculatum	ISP2	*Microbacterium hydrothermal* sp. 13	98.16
2017.05	2	Kandelia candel	HL2	*Micrococcus yunnanensis* sp. 14	99.56

### Phylogenetic analysis

**Figure 1:**
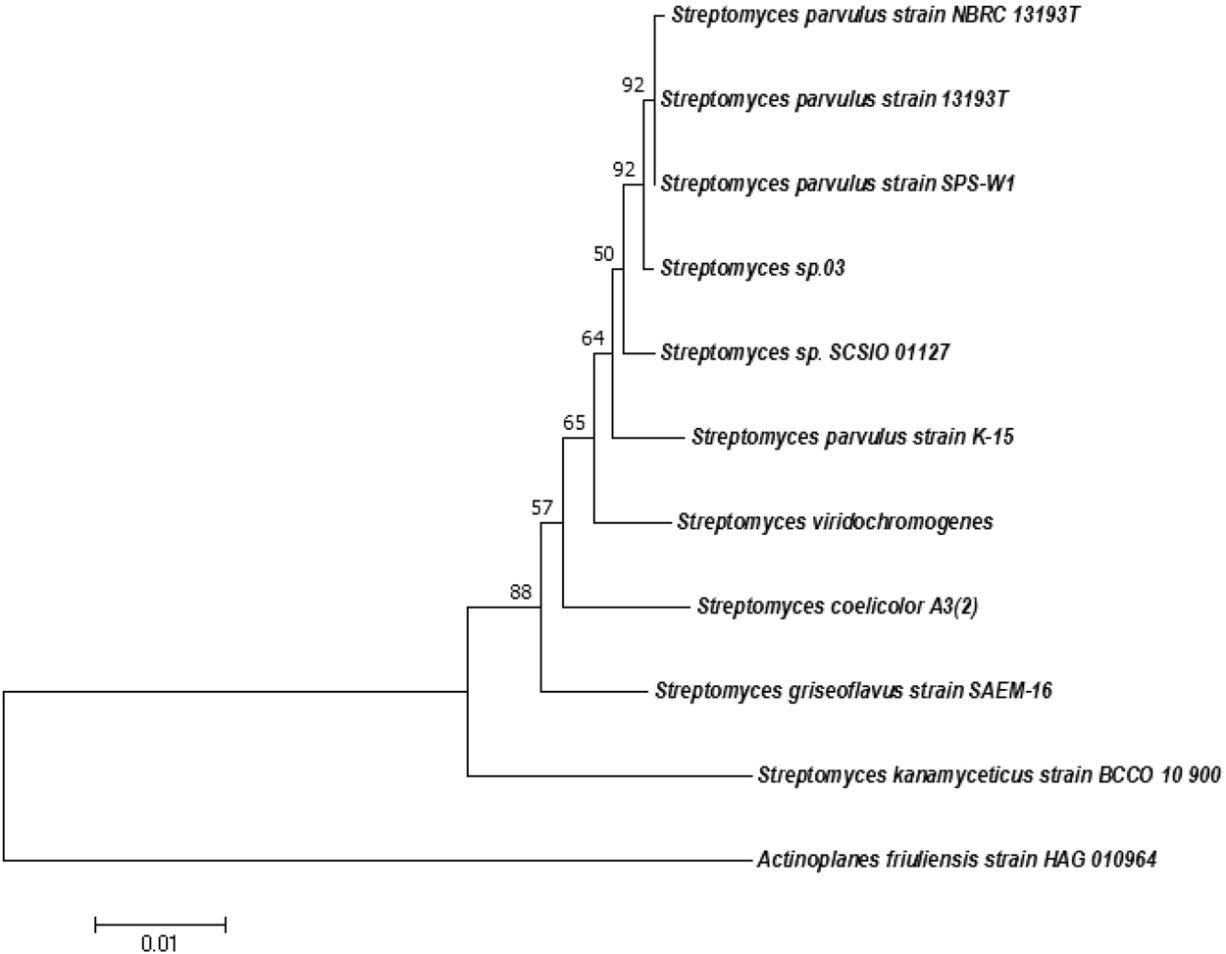
Comparison of 10 16S rRNA sequences from *Streptomyces parvulus* strain with other orthologous sequences. The neighbor-joinging method was used to construct the phylogenetic tree. The number of bootstrap replications was set to 1000.

Phylogenetic analysis based on 16S rRNA sequences showed that *Streptomyces* sp.03. and the other models of *Streptomyces parvulus* strain except for *Streptomyces parvulus* strain K-15, were located on the same branch of the tree, which suggests that the isolated strains should have a close relationship with them (Fig. 1). The *Streptomyces* sp.03 was also closer to *Streptomyces viridochromogenes, Streptomyces* sp. SCSIO 01127 and *Streptomyces coelicolor* A3(2), but have a relatively long distance with *Streptomyces kanamyceticus* strain BCCO 10900, *Streptomyces griseoflavus* strain SAEM-16 and *Actinoplanes friuliensis* strain HAG 010964. Therefore, the results showed that *Streptomyces* sp.03 have a close genetic distance with *Streptomyces parvulus* strain, for which the name *Streptomyces parvulus* strain 03 was proposed.

### Genome assembly and annotation

**Figure 2:**
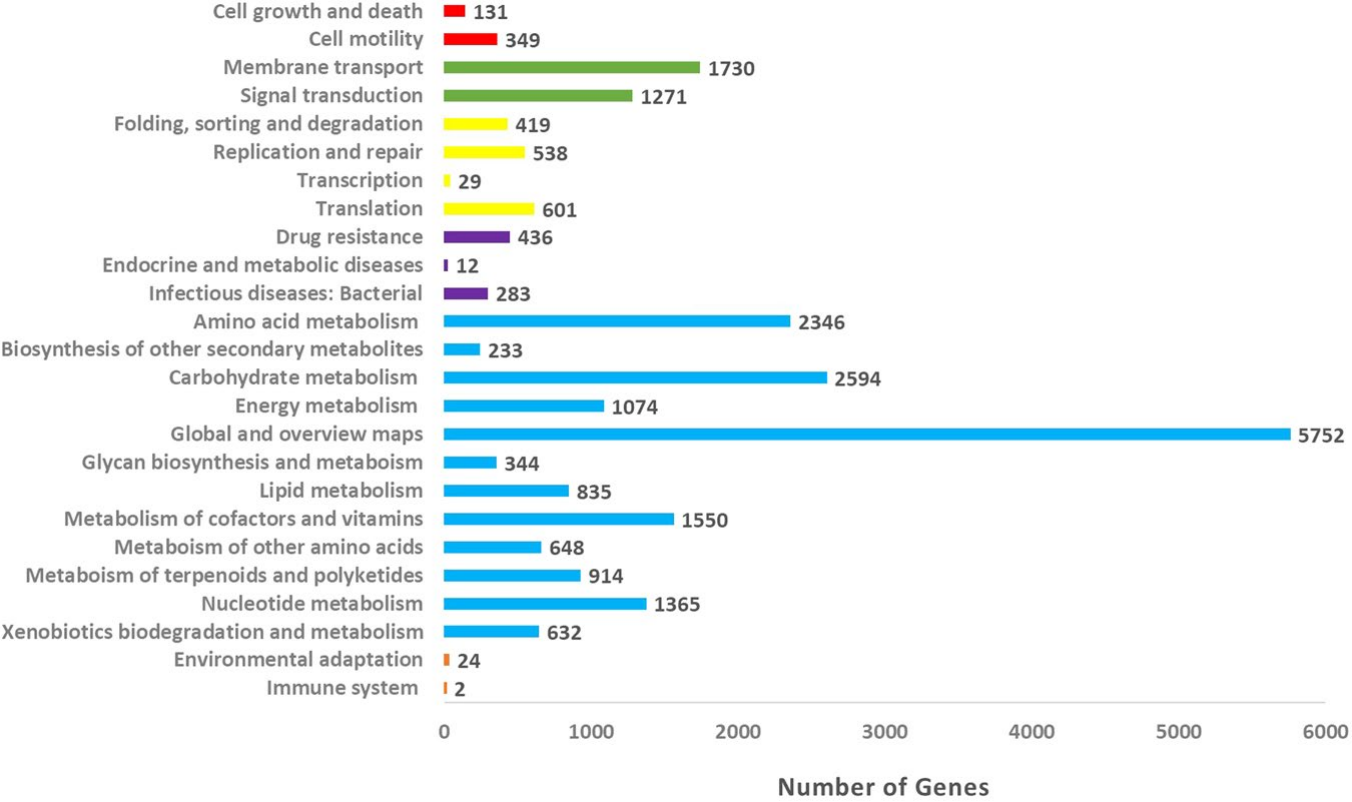
KEGG functional classification of proteins in the *Streptomyces parvulus* strain 03 genome. The distributions of the predicted proteins were assigned by the KEGG database. The number of sequences assigned to each sub-category of the five top KO categories, namely cellular process (red), environmental information processing (green), genetic information processing (yellow), human diseases (purple), metabolism (blue) and organismal systems (orange), were calculated and displayed.

One of the isolated strains, *Streptomyces parvulus* strain 03 has 99.13% 16S rRNA gene similarity to the sequence of *Streptomyces parvulus* strain 2297. *Streptomyces parvulus*, a species firstly identified from a soil sample by Waksman in 1940, was reported to produce several bioactive compounds including Actinomycin D, Borrelidin, Manumacin A, B and C, and Oleficin^36^. Whole-genome sequencing of the *Streptomyces parvulus* strain 03 produced a total of 6,147,555 reads and 118 scaffolds (Table 3). De novo assembly was done using IDBA, yielding a total consensus of 8,348,559 bp (72.28% G + C content) distributed within one main scaffold with an average length of 5,539 bp. The genome sequences were annotated by comparison with the KEGG (Kyoto Encyclopedia of Genes and Genomes) database using predicted genes. Within the *Streptomyces parvulus* strain 03, a total of 7,654 protein-encoding genes were conserved in strains, 72 tRNA and 14r RNA were predicted, the average CDS length was 959 bp and the coding density was about 87.91%. The predicted proteins were assigned by the KEGG database, and the top four categories in the KEGG functional classification were “global and overview map, carbohydrate metabolism, amino acid metabolism and membrane transport” (Fig. 2).

**Table 3:** General features of the genomes of isolated *Streptomyces parvulus* strain 03.

Sample	*Streptomyces parvulus* strain 03
Length (bp)	8348559
Coding density (%)	87.91%
Secondary metabolite biosynthetic gene coverage (%)	17.48%
Average CDS length (bp)	958.8801934
No. of protein-coding genes	7654
No. of tRNA genes	72
No. of reads	6147555
No. of contigs	118
No. of scaffolds	118
No. of rRNA	14
GC content	72.28

### Genome mining of *Streptomyces parvulus* strain 03

A total of 109 secondary metabolites biosynthesis gene clusters were identified in the *Streptomyces parvulus* strain 03, which were predicted for terpene, T1pks (type I polyketide synthases), T2pks (type II polyketide synthases), T3pks (type III polyketide synthases), Nrps (non-ribosomal peptide), indole, siderophore, bacteriocin, thiopeptide, phosphonate, lanthipeptide, ectoine, butyrolactone, T3pks-Nrps, T1pks-Nrps and other products (Supplementary Table S2). In which, 22 gene clusters show the similarity more than 20% (Table 4).

**Table 4:** Overview of 22 secondary metabolites of biosynthetic gene clusters which show the similarity more than 20% of *Streptomyces parvulus* strain 03 detected by anti-SMASH.

Cluster	Type	From	To	Most similar known cluster	Reference strain	Accesion number
Cluster 11	Indole	96830	130105	Spore pigment biosynthetic gene cluster (25% of genes show similarity)	*Streptomyces avermitilis*	AB070937.1
Cluster 12	Terpene	240198	300413	Isorenieratene biosynthetic gene cluster (100% of genes show similarity)	*Streptomyces subsp. griseus* NBRC 13350	AP009493.1
Cluster 13	Terpene	324861	345919	2-methylisoborneol biosynthetic gene cluster (100% of genes show similarity)	*Streptomyces subsp. griseus* NBRC 13350	AP009493.2
Cluster 28	Terpene	60002	81015	Albaflavenone biosynthetic gene cluster (100% of genes show similarity)	*Streptomyces coelicolor* A3(2)	AL645882.2
Cluster 30	T2pks	147737	190285	Spore pigment biosynthetic gene cluster (66% of genes show similarity)	*Streptomyces avermitilis*	AB070937.1
Cluster 31	Nrps	1	37645	Laspartomycin biosynthetic gene cluster (23% of genes show similarity)	*Streptomyces viridochromogenes* strain ATCC 29814	HM756254.1
Cluster 35	Bacteriocin	27882	45218	Informatipeptin biosynthetic gene cluster (42% of genes show similarity)	*Streptomyces viridochromogenes* DSM 40736	GG657757.1
Cluster 44	Melanin-saccharide	61331	98809	Melanin biosynthetic gene cluster (80% of genes show similarity)	*Streptomyces avermitilis*	AB070939.1
Cluster 45	Siderophore	153416	169772	Desferrioxamine B biosynthetic gene cluster (100% of genes show similarity)	*Streptomyces coelicolor* A3(2)	AL645882.2
Cluster 46	Putative	193692	208207	PM100117/PM100118 biosynthetic gene cluster (52% of genes show similarity)	*Streptomyces caniferus* strain GUA-06-06-006A	LN997801.1
Cluster 53	Nrps	62527	122641	SCO-2138 biosynthetic gene cluster (71% of genes show similarity)	*Streptomyces coelicolor* A3(2)	NC_003888.3
Cluster 59	Putative	166180	180695	Kanamycin biosynthetic gene cluster (53% of genes show similarity)	*Streptomyces kanamyceticus* strain NBRC 13415	AB254080.2
Cluster 62	Nrps	37781	88700	Coelichelin biosynthetic gene cluster (90% of genes show similarity)	*Streptomyces coelicolor* A3(2)	AL645882.2
Cluster 65	Nrps	1	34293	Friulimicin biosynthetic gene cluster (48% of genes show similarity)	*Actinoplanes firuliensis*	AJ488769.3
Cluster 72	Terpene	1	18738	Hopene biosynthetic gene cluster (69% of genes show similarity)	*Streptomyces coelicolor* A3(2)	AL645882.2
Cluster 74	T1pks-Otherks	79979	128036	Arsenopolyketides biosynthetic gene cluster (87% of genes show similarity)	*Streptomyces lividans* 1326	NZ_CM001889.1
Cluster 75	Lantipeptide	129046	144506	SapB biosynthetic gene cluster (100% of genes show similarity)	*Streptomyces coelicolor* A3(2)	AL645882.2
Cluster 81	Ectoine	20432	30830	Ectoine biosynthetic gene cluster (100% of genes show similarity)	*Streptomyces chrysomallus*	AY524544.1
Cluster 87	Putative	82110	104815	Glycopeptidolipid biosynthetic gene cluster (20% of genes show similarity)	*Mycobacterium avium* strain 2151	AF143772.2
Cluster 93	T3pks-Nrps	1	46565	Lobophorin biosynthetic gene cluster (28% of genes show similarity)	*Streptomyces* sp. FXJ7.023	JX306680.1
Cluster 94	Other	357	63840	Lomaiviticin biosynthetic gene cluster (60% of genes show similarity)	*Salinispora pacifica* strain DPJ-0016	KF731828.1
Cluster 98	Putative	2849	21666	Frankiamicin biosynthetic gene cluster (21% of genes show similarity)	*Frankia* sp. EAN1pec	CP000820.1

The analysis showed that this *Streptomyces parvulus* strain 03 may contain at least 14 kinds of PKS and NRPS gene clusters, among which 3 gene clusters were novel. The core structures of several gene clusters can be identified, which prediction based on assumed PKS/NRPS collinearity and tailoring reactions not taken into accound. In the present results, seven of the PKS and NRPS gene clusters related to antibiotics compounds, including friulimicin, lobophorin, laspartomycin, colabomycin, borrelidin, pristinamycin and kanamycin (Table 5).

**Table 5:** Overview of 14 secondary metabolites of biosynthetic PKS/NRPS gene clusters of *Streptomyces parvulus* strain 03 detected by anti-SMASH.

From	To	Most similar known cluster	Reference strain	Accesion number
1	46565	Lobophorin biosynthetic gene cluster (28% of genes show similarity)	*Streptomyces* sp. FXJ7.023	JX306680.1
147737	190285	Spore pigment biosynthetic gene cluster (66% of genes show similarity)	*Streptomyces avermitilis*	AB070937.1
79979	128036	Arsenopolyketides biosynthetic gene cluster (87% of genes show similarity)	*Streptomyces lividans* 1326	NZ_CM001889.1
1	13303	Polyoxypeptin biosynthetic gene cluster (10% of genes show similarity)	*Streptomyces* sp. MK498-98F14	KF386858.1
117708	158023	Borrelidin biosynthetic gene cluster (16% of genes show similarity)	*Streptomyces parvulus* Tu4055	AJ580915.1
1	5481	Lasalocid biosynthetic gene cluster (13% of genes show similarity)	*Streptomyces lasaliensis* strain NRRL 3382 R	FM173265.1
1	37645	Laspartomycin biosynthetic gene cluster (23% of genes show similarity)	*Streptomyces viridochromogenes* strain ATCC 29814	HM756254.1
62527	122641	SCO-2138 biosynthetic gene cluster (71% of genes show similarity)	*Streptomyces coelicolor* A3(2)	NC_003888.3
37781	88700	Coelichelin biosynthetic gene cluster (90% of genes show similarity)	*Streptomyces coelicolor* A3(2)	AL645882.2
75698	120023	Phosphonoglycans biosynthetic gene cluster (3% of genes show similarity)	*Glycomyces* sp. NRRL B-16210	KJ125437.1
1	34293	Friulimicin biosynthetic gene cluster (48% of genes show similarity)	*Actinoplanes firuliensis*	AJ488769.3
1	6035	Pristinamycin biosynthetic gene cluster (5% of genes show similarity)	*Streptomyces pristinaespiralis* strain Pr11	FR681999.1
1	6568	Marformycins biosynthetic gene cluster (12% of genes show similarity)	*Streptomyces drozdowiczii* strain SCSIO 10141	KP715145.1
1	45179	Colabomycin biosynthetic gene cluster (18% of genes show similarity)	*Streptomyces aureus* strain SOK1/5-04	KF850685.1

The coelinchelin biosynthesis gene cluster of *Streptomyces parvulus* strain 03 contains 11 ORFs (open reading frame), and 1 catalytic domain related to the non-ribosomal peptide synthetase (Supplementary Fig. S6). In the genome of *Streptomyces parvulus* strain 03, core biosynthetic genes were annotated to enterobactin/ferric enterobactin esterase and AMP-dependent synthetase and ligase, additional biosynthetic genes related to metallo-beta-lactamase family protein, lysine/ornithine N-monooxygenase, methionyl-tRNA formyltransferase and glycerol kinase; some transport-related genes and regulatory genes also can be found in the *Streptomyces parvulus* strain 03 genomes. In addition, coelinchelin biosynthesis clusters of *Streptomyces parvulus* strain 03 have a high homology (90%) to the existing cluster of *Streptomyces coelicolor* A3(2), with respect to the similar amino acid sequence of 85% identity.

Another NRPS-type gene cluster contains 28 ORFs and is responsible for friulimicin biosynthesis (Supplementary Fig. S7). In this gene cluster, core and additional biosynthetic genes were annotated to condensation domain-containing protein, phosphopantetheine-binding domain-containing protein, AMP-dependent synthetase and ligase, acyl-CoA dehydrogenase, dioxygenase TauD/TfdA, arginosuccinate lyase/adenylosuccinate lyase and cysteine synthase. Friulimicin biosynthetic cluster is also composed of numerous proteins of unknown function; however, the amino acid sequence showed 75% identity and 48% gene similarity. Therefore, *Streptomyces parvulus* strain 03 have high potential for producing a friulimicin-analog.

Furthermore, laspartomycin and pristinamycin biosynthetic gene clusters were found in the *Streptomyces parvulus* strain 03. However, these two NRPS-type biosynthetic gene clusters show only 23% and 5% gene similarity; comparison of the amino acid sequence between clusters in the genome of *Streptomyces parvulus* strain 03 and the laspartomycin biosynthetic gene cluster showed no similarity (Supplementary Fig. S4). In the genome of *Streptomyces parvulus* strain 03, only two ORFs of core biosynthetic genes were related to laspartomycin biosynthesis.

In addition to the diverse NRPS gene cluster, *Streptomyces parvulus* strain 03 also harbors the PKS pathway: type 1 PKS and type 3 PKS were found in the genome of *Streptomyces parvulus* strain 03.

The T1PKS gene cluster is composed of 38 ORFs, of which 5 encode key T1PKS modulators with 7 domains (Supplementary Fig. S2). Compared with the most-similar known cluster of *Streptomyces parvulus* Tu4055, this cluster of *Streptomyces parvulus* strain 03 has several unique ORFs, such as cytochrome P450 and short chain dehydrogenase/reductase SDR, which indicate that the strain isolated from mangrove may have the potential to synthesize an unknown compound or borrelidin-analog.

Another PKS pathway, the T3PKS gene cluster, is related to kanamycin synthesis (Supplementary Fig. S3). Comparison of both clusters indicates that numerous genes in this cluster cannot be annotated. There was only 1% gene similarity to the genome of *Streptomyces kanamyceticus*.

Except for the PKS and NRPS biosynthetic gene clusters, hybrid synthase was found in the genome of *Streptomyces parvulus* strain 03, including T1pks-Nrps and Butyrolactone-T2pks-Ladderane (Supplementary Fig. S5). The colabomycin biosynthesis gene cluster of *Streptomyces parvulus* strain 03 contains 44 ORFs, in turn containing 13 catalytic domains related to the non-ribosomal peptide synthetase. However, this cluster of *Streptomyces parvulus* strain 03 has more biosynthetic genes related to the production of antibiotics than the *Streptomyces aureus* strain SOK1/5-04 cluster. In the genome of *Streptomyces parvulus* strain 03, core biosynthetic genes were annotated to the condensation domain-containing proteins, thioesterases, beta-ketoacyl synthase, 3-oxoacyl, luciferase family protein, AMP-dependent synthetase and ligase and putative acyl carrier protein.

### Secondary metabolites identified by mass spectrometric analysis

**Figure 3:**
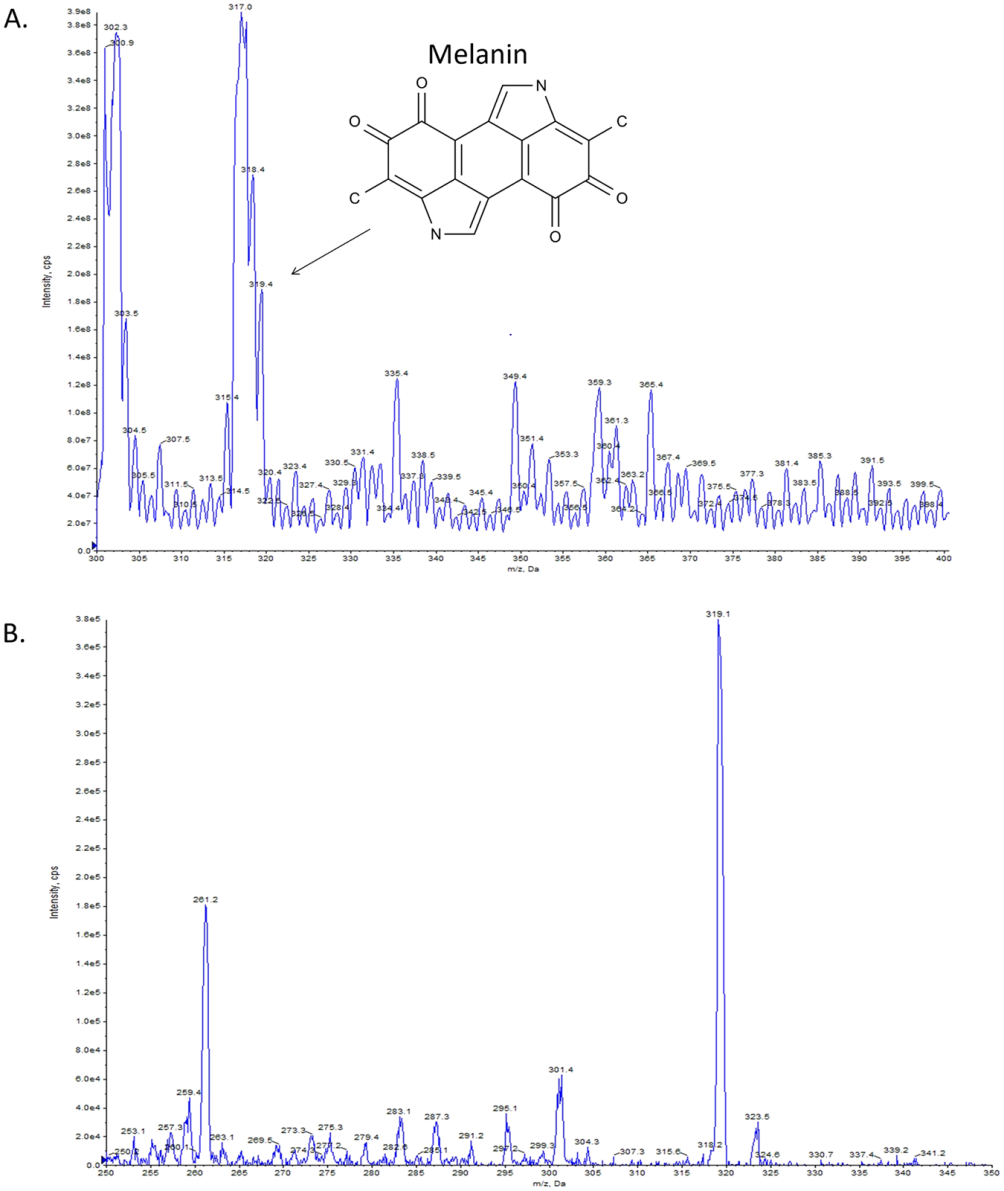
High-resolution LC-MS analysis of ethyl acetate (EA) extract of melanin in the fermentation broth of *Streptomyces parvulus* strain 03. The melanin ([M + H]^+^ at m/z 319.4 (**A**), in-source fragments at m/z 301.4, 291.2, 273.3, 263.1 and 261.2 (**B**)) was identified.

**Figure 4:**
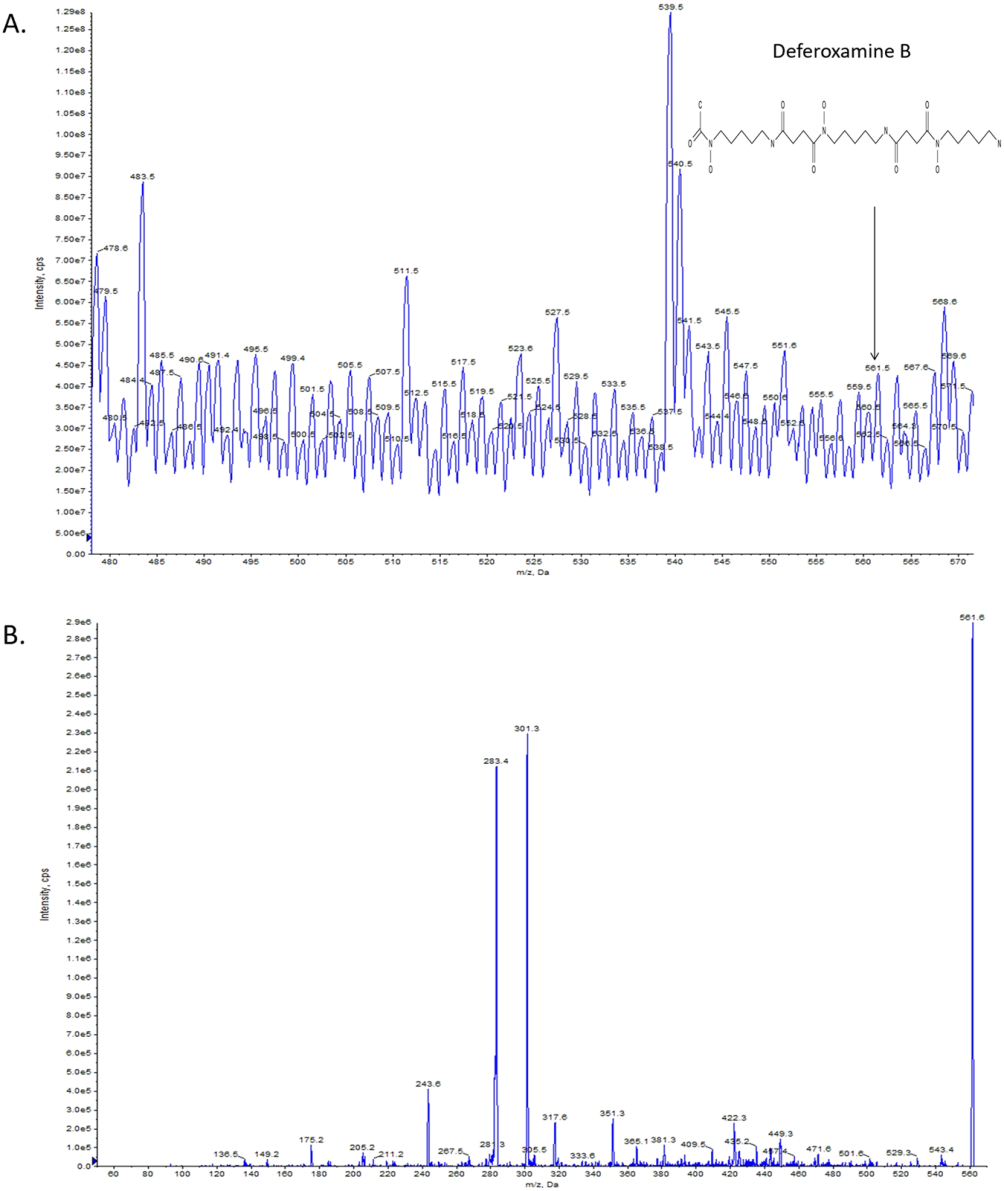
High-resolution LC-MS analysis of ethyl acetate (EA) extract of desferrioxamine B in the fermentation broth of *Streptomyces parvulus* strain 03. The deferoxamine B ([M + H]^+^ at m/z 561.5 (**A**), in-source fragments at m/z 243.6 (**B**)) was identified.

Fermentation studies were performed to test the hypothesis of whether the predicted secondary metabolites can be produced. Mass spectral data was obtained in positive mode. From the UPLC-MS profiles, the metabolic substances in the fermentation broth were identified according to the mass-to-charge ratio of molecular ions. Profiles of secondary metabolites present in isolates strain were compared with the genome mining data. In this case, two predicted secondary metabolites were identified in the extract, including melanin (Fig. 3, cluster 44, m/z = 319.4[M + H]^+^) and desferrioxamine B (Fig. 4, cluster 45, m/z = 561.5 [M + H^+^]).

## Discussions

Microorganisms with inherent physiological and functional diversity have been widely applied in agriculture, medicine, industry and environmental research^37^. Among the diverse industrially used microorganisms, actinomycetes are extremely important and are primarily recognized as a source of antibiotics^38^. Actinomycetes are a group of gram-positive filamentous bacteria with a high GC content, whose members have the ability to produce diverse secondary metabolites^22^. Nevertheless, in recent years, searching for new compounds from among many well-known actinomycetes strains isolated from different natural environments has become increasingly difficult^39^. This phenomenon can be explained due to frequent genetic exchanges between different species of bacteria^40,41^. Therefore, the search for new ecosystems in largely unexplored areas and unusual environments has become vital for the discovery of novel compounds and bacteria^42^. In our study, plant samples were collected from a mangrove ecosystem, at a tidal swamp in a tropical delta, and showed high potential for the discovery of new natural products. From the isolation results, 71 bacteria were isolated from plant samples, among which 14 isolates were identified as belonging to actinomycetes. As we know, actinomycetes produce numerous secondary metabolites for human pharmaceutical use such as antibiotics at which many of them were derived from genus of *Streptomyces*^43^. Previous research have proven that naturally occurring *Streptomyces* species was a promising source for antimicrobial agents^44^. A valuable study also have demonstrated that *Streptomyces parvulus* strain has wide-ranging antimicrobial activity, in particular effectively inhibiting the growth of *Pseudomonas putida, Salmonella typhi, Bacillus subtilis* and *Klebsiella pnuemoniae*^45^. Hence, identification of novel compounds in *Streptomyces parvulus* strain may be useful for fighting various pathogenic bacteria. Therefore, isolated *Streptomyces* sp.03 which have 99.13% similarity with *Streptomyces parvulus* was selected to identify the biosynthetic gene cluster and fermentation product.

Sequenced genomes can provide substantial evidence of rich secondary metabolic pathways. For example, regarding the biosynthetic pathway of kanamycin B and kanamycin A in *Streptomyces kanamyceticus*, it was recently shown that kanamycin A is derived from KanJ-and-KanK-catalyzed conversion of kanamycin B^46^ and genome mining of several actinobacteria has led to the elucidation of biosynthetic pathways of multiple bioactive compounds, including caboxamycin, geldanamycin, salinomycin and terpenoids^47–50^. According to the information obtained in our study, it is rational to identify the gene cluster involved in synthesis pathway of secondary metabolites by complete sequencing, assembly and annotation of the genome of *Streptomyces parvulus* strain 03 which isolated from the mangrove. The genome mining results showed that isolated *Streptomyces parvulus* strain 03 has the potential to produce diverse antibiotics, such as friulimicin, lobophorin, laspartomycin, colabomycin, borrelidin, pristinamycin and kanamycin. Recently, according to the improvement of prediction algorithm, anti-SMASH can predict around hundred gene clusters for *Streptomyces* strain. In our study, the genome was predicted to encode a total of 109 biosynthetic gene clusters, significantly higher than in other models of *Streptomyces parvulus* strain^51^. Compared to the previous report, complete genome sequence of the model *Streptomyces coelicolor* A3(2) revealed that more than 20 clusters coding for known or predicted secondary metabolites^21^. Engineered *Streptomyces avermitilis* host about twenty of the biosynthetic gene clusters for secondary metabolites^52^. In addition to that, some other *Streptomyces* strains have finished the genome sequencing. Such as the streptomycin-producing microorganism *Streptomyces griseus* IFO 13350 contains 34 gene clusters for the biosynthesis secondary metabolites^53^. Moreover, in previous studies, *Streptomyces parvulus* has been widely reported to produce actinomycin D, borrelidin (an angiogenesis inhibitor), manumycin A, B and C (antineoplastic agents) and oleficin (an antibacterial and antifungal agent)^54–56^. However, in our study comparing the rich genetic potential for secondary metabolites in the isolated *Streptomyces parvulus* strain 03 genome, only borrelidin was found in the biosynthetic gene cluster prediction. Moreover, these predicted compounds have never been identified and reported in any fermentation cultivation condition by *Streptomyces parvulus*^54^. Predicted gene clusters from the *Streptomyces parvulus* strain 03 were widely identified in other *Streptomyces* strains and non-*Streptomyces* strains, such as *Actinoplanes friuliensis*, *Streptomyces* sp. SCSIO 01127*, Streptomyces viridochromogenes, Streptomyces griseoflavus, Streptomyces pristinaespiralis*, and *Streptomyces kanamyceticus*^57–62^. Meanwhile, we can also observe that these mentioned species exhibited a relevant close relationship with the *Streptomyces parvulus* strain 03 in the phylogenetic tree. It is unquestionable, based on the analysis of the genome of *Streptomyces parvulus* strain 03 isolated from mangrove, that secondary metabolite production patterns are highly complex. Thus, genome sequencing results showed that bacteria will have adaptation strategies to cope with extreme environments^63,64^. Compared with normal strains, the genome of bacteria growing in extreme environments have a greater number of genes involved in various metabolic pathways^65^. Therefore, actinomycetes strains isolated from extreme environments, such as mangrove, with the support of DNA sequencing technology and bioinformatics may keep an unimagined potential to explore novel or more natural products.

In the fermentation experiment, we demonstrated that the isolated *Streptomyces parvulus* strain 03 can produce the secondary metabolites of melanin and desferrioxamine B via genome mining. This result highlights that most biosynthetic gene clusters are cryptic, at least under typical laboratory culture conditions^66^. As we know, secondary metabolites production of a microorganism are catalyzed by a series of enzyme-encoding genes^67^. The first NRPS-independent pathway of DKP biosynthesis was discovered in *Streptomyces noursei* in 2002, which showed that a probable tRNA (5-methylaminomethyl-2-thiouridylate)-methyltransferase and a probable NADP-specific glutamate dehydrogenase were involved in the DKP biosynthesis pathway^68^. Thus, genes involved in the production of metabolic substances might be silenced, as influenced by a specific regulation mechanism in the genome^69^, or the gene cluster may be un-expressed under normal cultivation conditions in the laboratory^70^.

In both identified compounds, melanin are generally black or brown pigments, which are frequently used in medicine, pharmacology and cosmetics preparation^71^. *Streptomyces* has a long history for the production of melanin, meanwhile, melanin formation in the *Streptomyces* species is the key feature for the classification of the *Streptomyces* group^72^. In our study, the saccharide-melanin was found from the *Streptomyces parvulus* strain. Up to now, there is no report suggesting that *Streptomyces parvulus* strain can produce saccharide-melanin. Hence, our study indicated that the *Streptomyces parvulus* strain have more biosynthesis potential waiting to be explored. In addition, desferrioxamine B can also be found in the fermentation broth, which is a specific iron complexing agent. It is available for clinical use as ‘Desferal’ (desferrioxamine B methanesulphonate) and has undergone extensive evaluation in the treatment of chronic iron overload states^73^. Previous study have determined that desferrioxamine E can be produced by *Streptomyces parvulus* CBS548.68, but no report suggesting that *Streptomyces parvulus* strain can produce desferrioxamine B^74^. Our study represents the first record of saccharide-melanin and desferrioxamine B produced from *Streptomyces parvulus* strain. Thus, using genome mining combined with chemical database searches and LC-MS screening can facilitate exploration of the biosynthetic potential of actinomycetes strains.

## Summary

In conclusion, our data showed that the mangrove is a good source for isolation of actinobacteria having a high number of secondary metabolites. At least two secondary metabolites of saccharide-melanin and desferrioxamine B were found in the fermentation broth by combining with whole-genome results. In the future, the application of efficient strategies to mine metabolite-encoding gene clusters in bacteria from extreme environments, while identifying the corresponding metabolites, presents an opportunity and challenge with respect to natural products and drugs discovery. Continued efforts towards culturing actinomycetes strains from mangrove would also represent a unique and promising means of discovering diverse secondary metabolites.

## Electronic supplementary material

Supplementary table S1

Supplementary table S2

Supplementary figure S1

Supplementary figure S2

Supplementary figure S3

Supplementary figure S4

Supplementary figure S5

Supplementary figure S6

Supplementary figure S7
