# Supplementary material for: Genome guided investigation of antibiotics producing actinomycetales strain isolated from a Macau mangrove ecosystem
Source: Sci Rep. 2018 Sep 24;8:14271. doi: 10.1038/s41598-018-32076-z (PMC6155160; doi:10.1038/s41598-018-32076-z)
Supplement: Supplementary file 4 — Supplementary figure S2 [file 41598_2018_32076_MOESM4_ESM.pdf]

Genome guided investigation of antibiotics producing actinomycetales strain isolated from a Macau mangrove ecosystem

Dini Hu[1]; Yan Chen[2]; Chenghang Sun[3]; Tao Jin[4]; Guangyi Fan[4]; Qiwen Liao[2]; Kai Meng Mok[1] and Ming-Yuen Simon Lee[2*]

[1] Faculty of Science and Technology, Department of Civil and Environmental Engineering, University of Macau, Macao, China;

[2] State Key Laboratory of Quality Research in Chinese Medicine and institute of Chinese Medical Sciences, University of Macau, Macao, China;

[3] Institute of Medicinal Biotechnology, Chinese Academy of Medical Science & Peking Union Medical College, Tiantanxili No 1, Beijing 100050, P.R. China;

[4] Beijing Genome Institute–Shenzhen, Shenzhen 518083, China.

Correspondence: Ming-Yuen Simon Lee, simonlee@umac.com

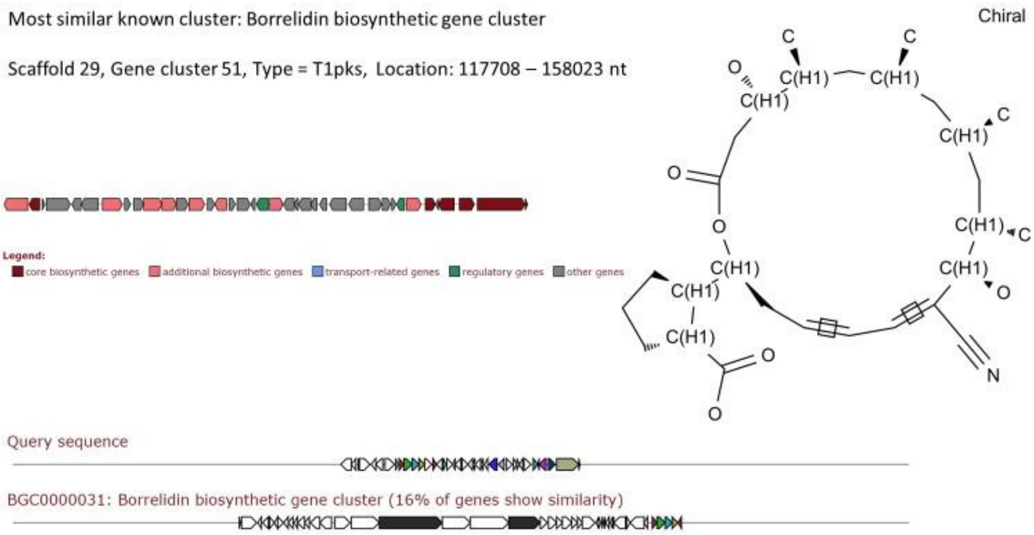

Supplementary figure S2. Proposed biosynthetic gene cluster of borrelidin-like compound in the *Streptomyces parvulus* strain 03. The most similar gene cluster from *Streptomyces parvulus* Tu4055 is shown, with related genes drawn in the same color to highlight inter-cluster rearrangements.
